# Appearance of a sore throat caused by the SARS‐CoV‐2 Omicron variant

**DOI:** 10.1002/jgf2.589

**Published:** 2022-11-07

**Authors:** Takeshi Yamashita, Takahiko Fukuchi, Hitoshi Sugawara

**Affiliations:** ^1^ Division of General Medicine, Department of Comprehensive Medicine 1, Saitama Medical Center Jichi Medical University Saitama City Japan

## Abstract

A 37year old Japanese man experienced severe sore throat. He was infected by the Omicron variant of SARSCoV2. The posterior pharyngeal wall in the left showed closely aggregated multiple milletsized white spots with surrounding redness.

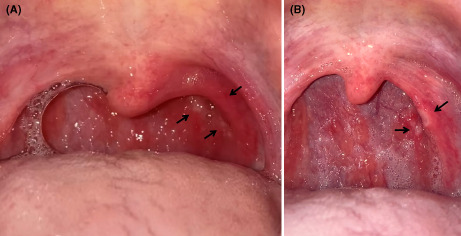

A 37‐year‐old Japanese man, who underwent tonsillectomy at the age of 24 years, experienced severe sore throat and presented to our hospital the day after the pain started. On Day 3, the result of a reverse‐transcription polymerase chain reaction (RT‐PCR) test revealed that he was infected by the Omicron variant of severe acute respiratory syndrome coronavirus 2 (SARS‐CoV‐2). The patient reported fever (38.9°C) on Day 2, with no dyspnea or cough, and his saturation of percutaneous oxygen (SpO_2_) was 98% in ambient air. The patient had received the third dose of the Pfizer–BioNTech BNT162b2 mRNA vaccine 2 months previously. Although the patient took loxoprofen, he experienced worsening of soreness on the left side of his throat and reported that he was unable to swallow saliva. Furthermore, he reported that he had lost 3 kg because of the associated difficulty in eating. On Day 5, the posterior pharyngeal wall in the left (Figure 1A) showed closely aggregated multiple millet‐sized white spots with surrounding redness, which did not resemble the follicles associated with influenza. Influenza follicles are multiple, round, and hemispherical lymph follicles on the posterior pharyngeal wall.^1^ On Day 7, several of the spots coalesced to form small ulcers and an area of redness converted to an ulcer (Figure 1B). On Day 9, his symptoms improved, and he was able to return to work after on Day 10.

**FIGURE 1:**
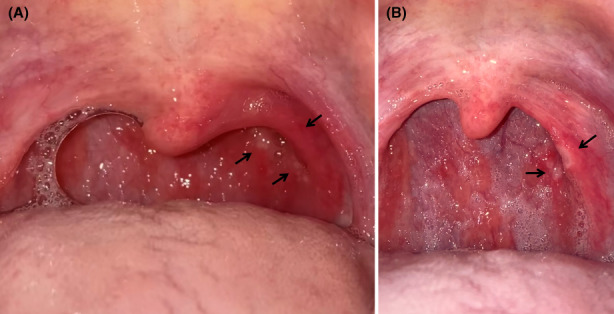
Photographs of the patient's posterior pharynx. (A) on Day 5, the posterior pharyngeal wall was covered with closely aggregated multiple millet‐sized white spots with surrounding redness (black arrows). (B) on Day 7, the spots coalesced into small ulcers

Sore throat is a common symptom associated with upper respiratory viral infections, such as influenza, measles, herpangina, infectious mononucleosis, and coronavirus disease (including COVID‐19). The incidence of severe sore throat episodes is reduced after tonsillectomy, but severe sore throat may still occur.^2^ The epidemiological burden of throat symptoms has considerably changed since the emergence of the Omicron SARS‐CoV‐2 variant, with a lower risk of chemosensory dysfunction and a higher risk of throat involvement.^3^ The Omicron variant appears to predominantly affect the upper airways and causes acute laryngitis without olfactory dysfunction. In some patients, the clinical manifestations are similar to those of epiglottitis.^4^ Although the patient underwent tonsillectomy and had received three doses of COVID‐19 vaccine, he still experienced a sore throat caused by SARS‐CoV‐2. The sore throat caused by SARS‐CoV‐2 appears not to have been related to the indication for tonsillectomy.

Reverse‐transcription polymerase chain reaction is the gold standard method for the diagnosis of COVID‐19. In the past few years, machine learning and artificial intelligence techniques have been proposed to help physicians diagnose “strep throat,”^5^ caused by Group A streptococcus infection. Closely aggregated multiple millet‐sized white spots and a painful posterior wall of the pharynx with surrounding redness may be a pathognomonic feature in the acute phase of the infection caused by the SARS‐CoV‐2 Omicron variant. If this hypothesis is correct, inspection of the pharynx may lead to a more rapid diagnosis of Omicron variant contaminations. Since it is unclear whether this finding is characteristic of the Omicron variant, further accumulation of cases is needed for confirmation.

## CONFLICT OF INTEREST

The authors have stated explicitly that there are no conflicts of interest in connection with this article.

## ETHICAL STATEMENT

Ethical approval by the institutional review board of Saitama Medical Center, Jichi Medical University, was not required at the authors' institution for this case report. The patient provided written informed consent for publication.

## PATIENT CONSENT STATEMENT

Written informed consent was obtained from the patient for publication of this clinical image.
